# Breastfeeding outpatient in primary care as an important action to promote breastfeeding: experience report

**DOI:** 10.1590/2317-1782/20232022234en

**Published:** 2024-05-27

**Authors:** Camila Dantas Martins, Carine Vieira Bicalho, Renata Maria Moreira Furlan, Amélia Augusta de Lima Friche, Andréa Rodrigues Motta

**Affiliations:** 1 Programa de Pós-graduação em Ciências Fonoaudiológicas, Universidade Federal de Minas Gerais – UFMG - Belo Horizonte (MG), Brasil.; 2 Departamento de Fonoaudiologia, Faculdade de Medicina, Universidade Federal de Minas Gerais – UFMG - Belo Horizonte (MG), Brasil.

**Keywords:** Breastfeeding, Health Education, Primary Health Care, Women's Health, Child Health, Infant

## Abstract

This paper describe a successful experience of promotion, prevention and support for breastfeeding developed by professionals from a basic health unit. This is a Breastfeeding Outpatient Clinic, implemented in a health center in Belo Horizonte, in August 2019. The Outpatient Clinic was established based on the perception of the unit's team that many mothers had difficulty breastfeeding, however, due to the work overload of this team, this assistance did not occur in a timely manner, resulting in early weaning. Initially, a meeting was held to sensitize the team on the breastfeeding indicators of the unit. Based on this knowledge, the implementation of a breastfeeding Outpatient clinic was proposed, aimed not only at dyad with difficulties in managing breastfeeding, but at all postpartum women in the area covered by that health center. A flow was created, through which it was established that all postpartum women who brought their children to carry out the heel prick test at the unit would be referred to the Breastfeeding Outpatient Clinic to perform this service. With the improvement of care, the users of the health center started to breastfeed for longer, which reflected in the improvement of the unit's indicators.

## INTRODUCTION

Exclusive breastfeeding (EBF) for up to 6 months and supplemented for up to 2 years or more is important in promoting and protecting maternal and child health. It is considered the most effective practice for reducing early childhood morbidity and mortality and can reduce neonatal mortality by 16.3% when started on the first day of life and by 22% if it occurs in the first hour after birth^(1)^. If all families practiced EBF until 6 months of their children's lives, followed by BF supplemented with other foods, it would be possible to save the lives of more than 800,000 children and 20,000 women per year worldwide^(2)^.

BF is much more than nourishment and brings countless benefits to the mother and newborn (NB)^(1)^. BF prevents diarrhea, respiratory infections, obesity, and chronic noncommunicable diseases in adulthood and promotes the child's intellectual development. Among BF mothers, it prevents breast cancer and postpartum obesity^(2)^.

These benefits are not restricted to the BF period but extend throughout life^(1)^. A 2019/2020 Brazilian survey found a 45.8% prevalence of EBF in children under 6 months old, with no statistically significant differences between the regions of the country^(2)^. However, the prevalence of EBF and continued BF in the first year of life, although significant, is still below that recommended by the World Health Organization (WHO)^(3-8)^.

Therefore, strategies are needed to promote BF and prevent BF difficulties at all levels of health care – especially in primary care, which is the gateway to health services. Primary care professionals must be prepared and qualified to provide comprehensive, humanized, quality, and timely assistance to postpartum women and newborns when they face any BF difficulty^(9)^.

BF is considered a challenging process for most women^(10-12)^. Influenced by emotional, cultural, social, and physiological/anatomical aspects, it is a complex activity that requires an adequate professional and family support network^(10)^.

Difficulties inherent to BF may involve the mother, the NB, or both, making it essential to evaluate them individually and together as early as possible. Studies highlight some variables that can interfere with BF and lead to early weaning, which include teenage mothers, low family income, employment, low educational attainment, single mothers, lack of previous experience with BF, lack of knowledge about the benefits of BF, lack of guidance on BF during prenatal care and in the maternity ward, and pain (most often due to inadequate BF technique). Furthermore, ankyloglossia and inadequate latch and positioning can also make BF difficult^(12)^.

Studies have described the positive impact of educational actions and interventions based on supporting women, both in the prenatal and postpartum periods, and such actions, when based on scientific evidence and adapted to local contexts, are essential to promote BF^(13-17)^.

Actions to promote BF must remove structural and social barriers that interfere with a woman's ability to breastfeed. According to some studies, EBF practice can be increased by 2.5 times, as long as combined interventions are implemented in health services and the community^(2-7)^. The analysis of EBF indicators in Brazil reinforces the need for investments in public policies and programs aimed at creating favorable environments that support women to breastfeed.

Thus, this report aimed to describe an action to promote, prevent, and support BF in a community health center (CHC).

## EXPERIENCE REPORT

This study is an experience report about care provided to postpartum women at the Vila Maria CHC, in the Northeast region of Belo Horizonte, Brazil.

Despite being characterized as an experience report, this study was submitted for consideration by the Ethics Committee of the Federal University of Minas Gerais, as it is part of a larger study involving human beings. It was approved under number 4.952.442.

Regarding management and central administration, Belo Horizonte is organized into nine Regional Administration Departments, called Health Districts. Their role is to coordinate the implementation of public urban, environmental, social, and health policies in their respective districts – i.e., the regions that encompass the geographic/territorial limits under each management. The Northeast Health District, to which Vila Maria CHC belongs, has 21 health centers. Vila Maria CHC provides comprehensive assistance to approximately 14,000 residents in the territory, including the neighborhoods of Jardim Vitória and Getsêmani.

**Figure 1:**
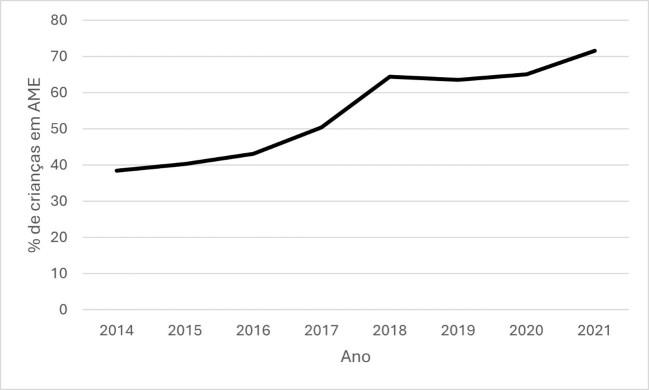
Exclusive breastfeeding indicators up to 4 months old at the Vila Maria health center from 2014 to 2021

Given this CHC’s low EBF indicators over the last few years (Figure 1), a team meeting held in January 2018 with representatives from all professional categories proposed the creation of a BF committee.

## IMPLEMENTATION OF THE CHC’S BF COMMITTEE

Following the example of existing committees in institutions that joined the Baby Friendly Hospital Initiative, the CHC’s BF committee should be responsible for developing actions in favor of maternal and child health, especially concerning BF and healthy complementary food for children under 2 years old, and breast milk donation at the CHC.

The committee had the following goals:

- Promoting optimal child growth and development;
- Helping reduce child mortality;
- Helping increase BF rates and good practices in complementary feeding for children under 2 years old;
- Integrating actions to encourage BF and promote healthy complementary feeding for children under 2 years old in the community;
- Raising awareness and training the CHC on “Breastfeeding, healthy complementary feeding up to 2 years old, and breast milk donation”.

This committee holds bimonthly meetings and was initially composed of a speech-language-hearing pathologist, a CHC manager, three community health workers, a nurse, two licensed practical nurses, a gynecologist, and more recently a nutritionist.

## THE PROPOSAL FOR AN EBF OUTPATIENT CLINIC

The discussions held by this committee led to the proposal for a BF outpatient clinic at Vila Maria CHC to promote comprehensive assistance to postpartum women and NBs. It would implement differentiated service, providing BF guidance and evaluation of postpartum women, NBs, and the mother-NB duo, identifying difficulties inherent to this process, and offering solutions to these problems, thus promoting BF.

Outpatient clinic refers to a multidisciplinary clinical care service, included in national public policies, and articulated with society to address the population's health problems. Hence, the team chose the name BF outpatient clinic to refer to care aimed at difficulties inherent to BF, provided by professionals specializing in BF.

### Flowchart for care at the BF outpatient clinic

**Figure 2:**
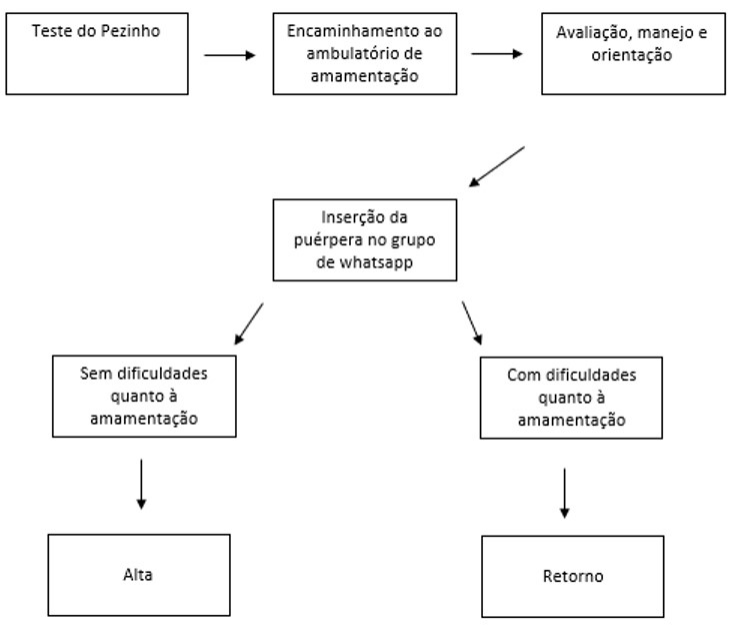
Flowchart of postpartum woman referral to the breastfeeding outpatient clinic at the Vila Maria health center

An internal flow was initially implemented at the CHC (Figure 2), through which postpartum women are referred to the said service. When they come to the CHC for the heel prick, an appointment is scheduled at the outpatient clinic, thus ensuring care for all postpartum women, not just those who report difficulties.

It is important to highlight that in 2020 and 2021, despite the COVID-19 pandemic, the flowchart for referral and care at the BF outpatient clinic was maintained, given that care for pregnant and postpartum women was considered a priority in Belo Horizonte.

### Contextualization of the sample

The clinic attended 204 breastfeeding women from August 2018 to January 2021. The postpartum women had a mean age of 33 years, and 50.0% of them had finished high school. The mean family income was up to two minimum wages. Most participants were multiparous (75.0%), all received prenatal care, and the preference was for normal delivery (83.3%). None of the breastfeeding women who participated in the study received prenatal BF guidance during the current pregnancy.

Of the postpartum women who participated in the study, 53.9% reported having some BF difficulty, including BF pain when associated with injured (83.4%) and engorged breasts (16.6%). Little milk was also reported by 16.6% of mothers. All complaints/difficulties mentioned were centered on the postpartum women. The most frequent complaint among postpartum women who participated in this study was BF pain (100%).

### Service operation

Care is provided by the CHC’s speech-language-hearing pathologist, who specializes in maternal and child health and is an international lactation consultant, with extensive experience in the area. Her schedule is fixed for this purpose, offering eight visits per week in two shifts, which meets the needs of the service. Postpartum women wait 1 week on average to be seen after scheduling an appointment. The other members of the committee participate in actions to promote and protect BF, such as pregnancy and childcare groups, waiting room activities, and continuing education for CHC employees.

A specific protocol was developed for the service, using as reference other ones validated in the literature, such as the breastfeeding observation and assessment protocol, recommended by UNICEF, and the LATCH breastfeeding assessment scale^(18,19)^. The consultations lasted an average of 1 hour, in which the medical history was surveyed through guided conversation and the mother and NB were assessed individually and together. The mother’s assessment verified the anatomy of the breast and nipple and the appearance of the breast tissue. The NB was assessed regarding feeding reflexes, protection, anatomy of the lingual frenulum, behavioral status, sucking pattern, sucking-swallowing-breathing coordination, rhythm, and anthropometric aspects, such as weight, length, and head circumference. The duo was assessed in terms of latch, the mother’s and NB’s positioning, and their bonding.

The mothers had their questions answered during consultations and were offered solutions to specific difficulties each one had. All consultations used the health counseling method^(13)^, which provides parents with adequate, scientifically-based information for them to make informed decisions and responsible choices. The guidance with parents addressed the following topics most often, according to their questions: correct latch and positioning, the importance of BF for NBs, postpartum women and their families, how to identify the newborn's satiety, how to maintain good milk production, the importance of free demand, how to burp the NB and for how long, and the risk of using nipple shields. If the mother did not have any BF difficulties, the NB was developing adequately, and her questions had been answered, she was discharged from the outpatient clinic but continued with other consultations according to the flow of the service. If any difficulties appeared later, they would be referred to the BF clinic again. If difficulties could not be solved in a single session, a return visit was scheduled within a maximum of 15 days.

Pain during breastfeeding (100.0%), associated with nipple trauma (83.4%), and breast engorgement (16.6%) were the most frequent complaints reported by postpartum women during the medical history survey, which corroborates with other studies published in the literature^(12)^. Once the mother’s difficulty was identified, it was clinically addressed as necessary to solve the problem. Studies have shown that clinical BF management in the first days after birth increases the prevalence of women breastfeeding at 1 month (OR: 1.49; 95% CI: 1.09-2.04) and EBF rates (OR: 1.71; 95% CI: 1.20-2.44)^(14-17)^.

A WhatsApp group was created to support postpartum women, including the professionals of the BF committee and breastfeeding women who attended the BF clinic. Hence, they could exchange information on motherhood and answer questions that might appear during BF.

## DISCUSSION

The characterization of the sample identified some factors that the literature describes as favorable to BF^(10,11)^, such as the mother’s age above 32 years, being multiparous, education level, normal delivery, and having prenatal care.

On the other hand, BF pain is one of the main causes of early weaning identified in the literature^(2-7,10,11)^. This pain may be related to breast engorgement, mastitis, breast candidiasis, nipple trauma, and so forth^(10,11)^. Professionals specializing in BF must identify causal factors and use strategies for continued breastfeeding. The main complaint reported among postpartum women in this study was BF pain, associated with nipple trauma, which agrees with the findings in the literature^(10,11)^.

The high turnover and overload of health center employees, the population’s lack of appreciation for health promotion initiatives, and some health professionals’ lack of awareness about the BF outpatient clinic are challenges that must be overcome. Health services and society in general faced further challenges between March 2020 and January 2021 due to the coronavirus pandemic, which may have impacted the number of NBs attending the CHC, resulting in absences from appointments scheduled at the BF clinic. Studies highlight that public policies had to be reorganized during the pandemic, requiring adjustments in their operations^(20,21)^.

Despite the challenges, numerous benefits can be described since the outpatient clinic was implemented in the service. The CHC's EBF indicator improved (Figure 1) and, more importantly, BF was prolonged, positively impacting the health of mothers and NBs, reducing their need for the services offered at the health center. This indicator, extracted from a specific SUS system from Belo Horizonte (called SIGRAH), results from a questionnaire administered by healthcare professionals during childcare consultations. Its questions were designed to construct BF indicators, as proposed by the WHO in 2008, subsequently revised in 2015, and endorsed by the Ministry of Health^(5)^.

Studies^(22,23)^ state that educational actions based on scientific evidence and adapted to local contexts are essential to promote BF. They include waiting room activities, educational material such as booklets, and sitting in a circle for conversation, through which the CHC also functions as a health education facility.

Research^(16,20,23)^ at different healthcare levels reports actions to promote, prevent, and support BF. However, we did not find reports like this in the literature, offering timely in-person attention in primary care, going beyond guidance, with professionals specializing in BF, with a fixed schedule to meet the needs inherent to BF – which indicates that this is an innovative action, especially within SUS, since many families would not have access to BF consultancy in the private sector.

This experience report presents limitations, such as its descriptive design with data on monitoring reported by the Municipal Health Department of Belo Horizonte. Nevertheless, it helps structure and propose actions to promote BF and prevent early weaning, which can be implemented in other SUS health centers in other cities as well.

Breast milk is indisputably known to be the best food for children, with positive impacts on maternal and child health. However, BF is not an innate skill; rather, it needs to be learned and developed. Therefore, mothers need assistance from health professionals and a support network to reduce the risks of early weaning.

## FINAL COMMENTS

The implementation of a BF clinic at SUS with qualified professionals to promote BF and prevent difficulties inherent to this process was a successful women's healthcare experience.

The Vila Maria CHC users received adequate and timely care, which improved the center's indicators over the years. This highlights the need to strengthen actions, policies, and programs to promote, protect, and support BF, as reported here.
