# Supplementary material for: Breastfeeding outpatient in primary care as an important action to promote breastfeeding: experience report
Source: Codas. 2024 May 27;36(3):e20220234. doi: 10.1590/2317-1782/20232022234en (PMC11166039; doi:10.1590/2317-1782/20232022234en)
Supplement: Translation

# Translation: Ambulatório de amamentação na atenção básica como uma importante ação de promoção ao aleitamento materno: relato de experiência

**DOI:** 10.1590/2317-1782/20232022234pt

**Authors:** Camila Dantas Martins, Carine Vieira Bicalho, Renata Maria Moreira Furlan, Amélia Augusta de Lima Friche, Andréa Rodrigues Motta

**Affiliations:** 1 Programa de Pós-graduação em Ciências Fonoaudiológicas, Universidade Federal de Minas Gerais – UFMG - Belo Horizonte (MG), Brasil.; 2 Departamento de Fonoaudiologia, Faculdade de Medicina, Universidade Federal de Minas Gerais – UFMG - Belo Horizonte (MG), Brasil.

**Keywords:** Aleitamento Materno, Educação em Saúde, Atenção Primária à Saúde, Saúde da Mulher, Saúde da Criança, Recém-nascido

## Abstract

O presente estudo tem por objetivo descrever uma experiência de promoção, prevenção e apoio ao aleitamento materno desenvolvida por profissionais de uma unidade básica de saúde. Trata-se de um ambulatório de amamentação, implementado em um centro de saúde, em Belo Horizonte, em agosto de 2019. O ambulatório foi instituído a partir da percepção da equipe da unidade de que muitas mães tinham dificuldade com o processo de amamentação, no entanto, em função da sobrecarga de trabalho desta equipe, esta assistência não ocorria em tempo hábil, resultando no desmame precoce. Inicialmente realizou-se uma reunião para sensibilização da equipe sobre os indicadores de aleitamento materno da unidade. A partir desse conhecimento foi proposta a implementação de um ambulatório de amamentação, destinado não apenas às puérperas com dificuldade no manejo do aleitamento materno, mas à todas da área de abrangência daquela Unidade Básica de Saúde (UBS). Para captação das puérperas, foi estabelecido um fluxo, por meio do qual ficou estabelecido que todas as puérperas que trouxessem seus filhos para realização do teste do pezinho na unidade, seriam encaminhadas ao ambulatório para a realização deste atendimento. Com a melhora da assistência, as usuárias da unidade básica de saúde passaram a amamentar por mais tempo, o que refletiu na melhora dos indicadores da unidade.

## INTRODUÇÃO

O aleitamento materno (AM) exclusivo até os seis meses e complementado até dois anos ou mais é um importante fator na promoção e proteção da saúde materno infantil, sendo considerado a prática mais eficaz para a redução da morbimortalidade na primária infância, podendo reduzir a mortalidade neonatal em 16,3% quando iniciada no primeiro dia de vida e em 22%, se ocorrer na primeira hora após o parto^(1)^. Se todas as famílias adotassem a prática de aleitamento materno exclusivo (AME) até os seis meses de vida dos seus filhos, seguido do aleitamento materno complementado com outros alimentos, seria possível salvar, anualmente, a vida de mais de 800 mil crianças e 20 mil mulheres no mundo^(2)^.

Amamentar é muito mais que nutrir e traz inúmeros benefícios para a dupla mãe-recém-nascido (RN)^(1)^. O aleitamento materno previne diarreias, infecções respiratórias, obesidade e doenças crônicas não transmissíveis na idade adulta, além de favorecer o desenvolvimento intelectual da criança. Entre as mães que amamentam previne câncer de mama e a obesidade pós-parto^(2)^.

Tais benefícios não se restringem apenas ao período da amamentação, mas estendem-se ao longo da vida^(1)^. De acordo com uma pesquisa realizada no Brasil entre 2019 e 2020, a prevalência de AME em menores de 6 meses foi de 45,8%, sem diferenças estatisticamente significativas entre as regiões do país^2^. Contudo, as prevalências de AME e de aleitamento materno continuado no primeiro ano de vida, embora expressivas, ainda estão aquém do preconizado pela Organização Mundial de Saúde (OMS)^(3-8)^.

Assim, se faz necessária a implementação de estratégias que promovam o aleitamento materno e previnam as dificuldades referentes à amamentação em todos os níveis de atenção à saúde, sobretudo na atenção básica, visto que essa é a porta de entrada para os serviços de saúde. Os profissionais da atenção primária devem estar preparados e capacitados para prestar uma assistência integral, humanizada, de qualidade e em tempo oportuno à puérpera e ao recém-nascido frente a qualquer dificuldade no aleitamento materno^(9)^.

A amamentação é considerada um processo desafiador para a maioria das mulheres^(10-12)^. É influenciada por aspectos emocionais, culturais, sociais e anátomo fisiológicos, sendo considerada uma atividade complexa que requer uma rede de apoio profissional e familiar adequada^(10)^.

As dificuldades inerentes ao processo de amamentação podem envolver a mãe, o recém-nascido ou ambos, tornando-se primordial a avaliação da puérpera, do recém-nascido e da díade, o mais precocemente possível. Estudos pontuam algumas variáveis que podem interferir na amamentação e levar ao desmame precoce. Dentre elas pode-se citar: ser mãe adolescente, ter baixa renda familiar, vínculo empregatício, baixa escolaridade, ser mãe solteira, ausência de experiência prévia com a amamentação, desconhecimento dos benefícios da amamentação, ausência de orientação sobre aleitamento no pré-natal e na maternidade, além da presença de dor (na maioria das vezes em função da técnica inadequada de amamentação). Além disso, anquiloglossia, pega e posicionamento inadequados, também podem dificultar o aleitamento materno^(12)^.

Estudos tem descrito o impacto positivo de ações educativas e intervenções baseadas no apoio à mulher, tanto no período pré-natal quanto no pós-parto e tais ações, quando baseadas em evidências científicas e adaptadas aos contextos locais, são essenciais para promover o AM^(13-17)^.

As ações que visam promover a amamentação devem remover barreiras estruturais e sociais que interferem na capacidade da mulher amamentar. De acordo com alguns estudos, é possível aumentar a prática do aleitamento materno exclusivo (AME) em 2,5 vezes, desde que sejam implementadas intervenções combinadas em serviços de saúde e na comunidade^(2-7)^. A análise dos indicadores do AME no Brasil reforça a necessidade de investimentos em políticas públicas e programas voltados à criação de ambientes favoráveis que apoiem as mulheres a amamentar.

Neste contexto, este relato teve como objetivo descrever uma ação de promoção, prevenção e apoio ao aleitamento materno desenvolvida em uma unidade básica de saúde.

## RELATO DA EXPERIÊNCIA

O presente estudo se refere a um relato de experiência acerca de atendimentos prestados a puérperas da Unidade Básica de Saúde (UBS) Vila Maria, localizado na regional Nordeste, no município de Belo Horizonte.

Apesar desse trabalho se caracterizar como um relato de experiência, o mesmo foi submetido à apreciação do Comitê de Ética da Universidade Federal de Minas Gerais por ser parte de um estudo maior que envolve seres humanos e foi aprovado sob o número 4.952.442

No âmbito da estruturação da gestão, além da administração central, o município de Belo Horizonte está organizado em nove Secretarias de Administração Regional, denominadas Distritos Sanitários. Essas têm a função de coordenar as ações de implantação das políticas públicas urbanas, ambientais, sociais e de saúde em suas respectivas circunscrições, ou seja, os territórios referentes aos limites geográficos/territoriais relativos a cada gerência. O Distrito Sanitário Nordeste, do qual a UBS Vila Maria faz parte, é constituído por 21 centros de saúde. O Vila Maria garante assistência integral à uma população de aproximadamente 14.000 moradores referenciados no território, que abrange os bairros Jardim Vitória e Getsêmani.

Mediante os baixos indicadores dessa unidade para aleitamento materno exclusivo ao longo dos últimos anos (Figura 1), em janeiro de 2018, foi realizada uma reunião de equipe, com representantes de todas as categorias profissionais, sendo então proposta a criação de um comitê de aleitamento materno.

**Figura 1:** Indicadores de aleitamento materno exclusivo, até os 4 meses de vida, no Centro de Saúde Vila Maria, de 2014 a 2021

## A IMPLEMENTAÇÃO DO COMITÊ DE ALEITAMENTO MATERNO NA UBS

A exemplo dos comitês que já existiam nas instituições que aderiram à Iniciativa Hospital Amigo da Criança, o comitê de aleitamento materno da UBS, deveria ficar responsável por desenvolver na unidade de saúde ações em prol da saúde materno infantil, sobretudo no que tange à amamentação e alimentação complementar saudável para menores de 2 anos e Doação de leite humano (LH) na UBS.

São objetivos do comitê:

- Promover o crescimento e desenvolvimento ótimos da criança;
- Contribuir para a redução da mortalidade infantil;
- Contribuir para o aumento dos índices de aleitamento materno e boas práticas na alimentação complementar para menores de 2 anos;
- Integrar as ações de estímulo ao aleitamento materno e de promoção de alimentação complementar saudável para menores de 2 anos na comunidade;
- Sensibilizar e Capacitar a Unidade Básica no tema “Aleitamento Materno, Alimentação Complementar Saudável até 2 anos de idade e Doação de LH.

Este comitê realiza reuniões bimestrais e foi composto inicialmente por um fonoaudiólogo, um gestor da unidade básica de saúde, três agentes comunitários de saúde, um enfermeiro, dois técnicos de enfermagem, um ginecologista e mais recentemente por um nutricionista.

## A PROPOSTA DO AMBULATÓRIO DE AMAMENTAÇÃO

Das discussões realizadas por esse comitê, surgiu a proposta do Ambulatório de Amamentação na UBS Vila Maria para promover uma assistência integral às puérperas e recém-nascidos, por meio da implementação de um serviço diferenciado que não se restringisse apenas a orientações sobre amamentação, mas a avaliação da puérpera, do recém-nascido e da dupla mãe-RN, identificar dificuldades inerentes a esse processo e propor soluções para esses problemas, promovendo assim o aleitamento materno.

Entendemos que o termo ambulatório se refere a um serviço de atendimento clínico multiprofissional, inserido nas políticas públicas nacionais e articulado com a sociedade para o enfrentamento dos problemas de saúde da população. O termo “ambulatório de amamentação” foi escolhido pela equipe para se referir ao atendimento voltado às dificuldades inerentes à amamentação, realizado por profissional especialista em amamentação.

### Fluxograma para atendimento no ambulatório de amamentação

Inicialmente foi implementado um fluxo interno da unidade (Figura 2), por meio do qual as puérperas são encaminhadas ao referido serviço. Quando a puérpera comparece a UBS para realização do teste do pezinho, é agendada uma consulta no Ambulatório. Dessa maneira, o atendimento é garantido a todas as puérperas e não apenas àquelas que relatam dificuldades.

**Figura 2:** Fluxograma para encaminhamento das puérperas ao Ambulatório de amamentação do Centro de Saúde Vila Maria

É importante ressaltar, que nos anos de 2020 e 2021, apesar da pandemia da covid-19, o fluxograma para encaminhamento e o atendimento no ambulatório de amamentação foi mantido, visto que no município de Belo Horizonte, o atendimento às gestantes e puérperas foi considerado prioritário.

### Contextualização da amostra

No período compreendido entre agosto de 2018 a janeiro de 2021 foram atendidas 204 lactantes. As puérperas apresentaram idade média de 33 anos e 50,0% cursaram o ensino médio completo. A renda familiar média foi de até dois salários-mínimos. A maioria das participantes era multípara (75,0%), todas realizaram pré-natal e a via de parto preferencial foi a normal (83,3%). Nenhuma das lactantes que participaram do estudo recebeu orientações sobre amamentação no pré-natal na gestação atual.

Das puérperas que participaram do estudo, 53,9% referiram ter alguma dificuldade para amamentar. Dentre tais dificuldades foram referidas dor para amamentar, associada a mamas feridas (83,4%) e mamas ingurgitadas (16,6%). Pouco leite também foi referido por 16,6% das mães. Todas as queixas/dificuldades referidas estavam centradas na puérpera. A queixa mais frequente, entre as puérperas que participaram deste estudo, foi dor durante a amamentação (100%).

### Funcionamento do serviço

Os atendimentos são realizados pela fonoaudióloga da UBS, que é especialista em saúde materno infantil, consultora internacional de lactação, com vasta experiência na área. A profissional tem agenda protegida para este fim e oferece oito vagas por semana, em dois turnos, sendo o número de vagas ofertadas, suficiente para a demanda do serviço. As puérperas esperam em média uma semana para serem atendidas a partir da marcação da consulta. Os demais membros do comitê estão envolvidos em ações de promoção e proteção ao aleitamento tais como grupos de gestante e de puericultura, ações em sala de espera e atividades voltadas à educação permanente dos funcionários da UBS.

Foi elaborado um protocolo específico para o serviço, utilizando-se como referência outros protocolos validados na literatura, como o protocolo de observação e avaliação da mamada, preconizado pela Unicef e a escala da avaliação da amamentação LATCH^(18,19)^. Os atendimentos duraram em média, uma hora e envolveram anamnese, por meio de conversa dirigida, avaliação da puérpera, do RN e da dupla. A avaliação da puérpera constou da avaliação da anatomia da mama, do mamilo e aspecto do tecido mamário. Quanto ao recém-nascido foram avaliados os reflexos de alimentação, proteção, anatomia do frênulo lingual, estado comportamental, padrão de sucção, coordenação entre sucção/deglutição e respiração, ritmo e aspectos antropométricos como peso, comprimento e perímetro cefálico. No que se refere à dupla, foi avaliada a pega, o posicionamento da puérpera, do recém-nascido e vínculo.

Durante o atendimento, as puérperas tiveram suas dúvidas sanadas e foram enfatizadas soluções das dificuldades apresentadas por cada mãe. É importante salientar que foi adotada a técnica de aconselhamento em saúde^(13)^ em todos os atendimentos realizados, visto que esta estratégia teve por objetivo fornecer aos pais informações adequadas, cientificamente embasadas, para que tomassem decisões informadas e fizessem escolhas responsáveis. As orientações mais frequentemente fornecidas aos pais baseadas em suas dúvidas foram sobre: pega e posicionamento corretos, importância do aleitamento materno para o RN, puérpera e toda família, como identificar a saciedade do recém-nascido, como manter uma boa produção láctea, importância da livre demanda, como colocar o RN para arrotar e por quanto tempo, e sobre o risco dos bicos artificiais. Caso a puérpera não apresente qualquer dificuldade inerente ao processo de amamentação, o desenvolvimento do RN esteja adequado e suas dúvidas tenham sido sanadas, esta receberá alta do ambulatório, porém seguirá com as demais consultas conforme fluxo do serviço. No entanto, caso surja qualquer dificuldade posteriormente, essa será novamente encaminhada ao ambulatório de amamentação. Nos casos em que as dificuldades não forem sanadas em um único atendimento, é agendado um retorno em, no máximo, 15 dias.

Identificou-se que dor para amamentar (100,0%), associada a traumas mamilares (83,4%) e ingurgitamento mamário (16,6%) foram as queixas mais frequentes relatadas pelas puérperas durante a anamnese, o que corrobora com outros estudos publicados na literatura^(12)^. A partir da identificação da dificuldade apresentada pela mãe, é realizado o manejo clínico necessário e o problema sanado. Estudos apontaram que o manejo clínico da amamentação quando realizado nos primeiros dias após o parto, aumentam a prevalência de mulheres em amamentação com um mês (OR:1,49; IC95%:1,09-2,04) e as taxas de aleitamento materno exclusivo (OR:1,71; IC95%:1,20-2,44)^(14-17)^.

Como apoio à puérpera foi criado um grupo de *WhatsApp* formado pelos profissionais que compõem o comitê de aleitamento e lactantes atendidas no ambulatório de amamentação, para que pudessem trocar informações referentes à maternidade, além de sanar possíveis dúvidas que possam surgir ao longo do processo de amamentação.

## DISCUSSÃO

A partir da caracterização da amostra foi possível perceber a presença de alguns fatores descritos na literatura como favoráveis à amamentação^(10,11)^, tais como: idade materna maior que 32 anos, ser multípara, escolaridade, parto normal e ter realizado pré-natal.

Já a dor para amamentar é uma das principais causas de desmame precoce apontadas pela literatura^(2-7,10,11)^. Essa dor pode estar relacionada ao ingurgitamento mamário, mastite, candidíase mamária, traumas mamilares, dentre outros fatores^(10,11)^. É importante que o profissional especialista em amamentação identifique o fator causal e utilize estratégias que possibilitem a continuidade da amamentação. No presente estudo, a principal queixa referida entre as puérperas foi a dor para amamentar, associada a traumas mamilares, o que concorda com os achados da literatura^(10,11)^.

A alta rotatividade e a sobrecarga dos funcionários do centro de saúde, a não valorização de ações que promovam a saúde por parte da população, a falta de sensibilização de alguns profissionais do serviço sobre a proposta do ambulatório de amamentação são considerados desafios que precisam ser superados. No período compreendido entre março de 2020 a janeiro de 2021, em função da pandemia pelo Coronavírus, foram adicionados novos desafios aos serviços de saúde e à sociedade como um todo. Esses desafios podem ter impactado no número de recém-nascidos frequentando as UBS, resultando em absenteísmo às consultas agendadas para o ambulatório de amamentação. Pesquisas ressaltaram que as políticas públicas precisaram ser reorganizadas durante a pandemia, com necessidade de adaptações na forma de operacionalização^(20,21)^.

Apesar dos desafios, podem ser descritos inúmeros benefícios desde que o ambulatório foi implementado no serviço. Houve melhora do indicador de AME da unidade (Figura 1), mas sobretudo a amamentação foi prolongada, o que repercute positivamente na saúde das mães e recém-nascidos, reduzindo a demanda pelos serviços oferecidos no centro de saúde. Esse indicador foi extraído por meio de um sistema próprio da rede SUS – BH, denominado SIGRAH. O indicador resulta de um questionário realizado pelos profissionais da área da saúde durante as consultas de puericultura. As perguntas provenientes do questionário foram elaboradas para permitir a construção dos indicadores de aleitamento materno propostos pela OMS em 2008, posterirormente revisados em 2015. Esses indicadores são endossados pelo Ministério da Saúde^(5)^.

Estudos^(22,23)^ referem que ações educativas, baseadas em evidências científicas, adaptadas aos contextos locais são essenciais para promover o aleitamento materno. Tais ações envolvem atividades em salas de espera, elaboração de material educativo como cartilhas, rodas de conversa, que contribuem para que as UBSs atuem também como equipamentos de educação em saúde.

Pesquisas^(16,20,23)^ em diversos níveis de atenção à saúde, relatam ações que visam promover, prevenir e apoiar a amamentação, no entanto, não encontramos na literatura, relatos como esse, à nível de atenção primária, no sentido de oferecer atendimento presencial, em tempo oportuno e não apenas orientações, com profissionais especialistas na área de amamentação, com agenda protegida, para atender demandas inerentes à amamentação, o que nos faz refletir que se trata de uma ação inovadora, sobretudo no âmbito do SUS, visto que muitas famílias não teriam acesso à uma consultoria de amamentação no setor privado.

Embora o presente relato de experiência apresente como limitações seu caráter descritivo com dados de monitoramento reportados pela Secretaria Municipal de Saúde de Belo Horizonte, é possível verificar sua contribuição com a estruturação e a proposição de ações de promoção do aleitamento materno e prevenção do desmame precoce que podem ser implementadas em outros Centros de Saúde da rede SUS, não apenas no município de Belo Horizonte.

É sabido que o leite materno é indiscutivelmente o melhor alimento para a criança, gerando impactos positivos na saúde materno-infantil. No entanto, amamentar não é uma habilidade inata, é preciso ser aprendida e desenvolvida e, portanto, é fundamental o suporte dos profissionais de saúde e da rede de apoio, visando reduzir os riscos do desmame precoce.

## COMENTÁRIOS FINAIS

Como prática para promover o aleitamento materno e prevenir dificuldades inerentes a este processo, a implementação de um ambulatório de amamentação no SUS, com profissionais qualificados, constituiu-se como uma experiência exitosa na atenção à saúde das mulheres.

Percebeu-se que as usuárias do centro de saúde Vila Maria foram assistidas de forma adequada e em tempo hábil, o que refletiu na melhora dos indicadores da unidade ao longo dos anos. Evidencia-se, assim, a necessidade do fortalecimento de ações, políticas e programas de promoção, proteção e apoio ao aleitamento materno, como a ação aqui relata.
